# Measuring Cup

**Published:** 1866-06

**Authors:** 


### MEASURING CUP.

This is a little appliance arranged by Dr. Shadoan, for determining the amount of rubber for any given case. By it the amount can be precisely determined, which in many cases is an important item. It consists of a small cup, with a glass tube and scale attached. The method of using it will be suggestive at first sight. We presume it can be obtained at any of the Dental Depots.

